# Long-term nitrogen deposition reduces the diversity of nitrogen-fixing plants

**DOI:** 10.1126/sciadv.adp7953

**Published:** 2024-10-18

**Authors:** Pablo Moreno-García, Flavia Montaño-Centellas, Yu Liu, Evelin Y. Reyes-Mendez, Rohit Raj Jha, Robert P. Guralnick, Ryan Folk, Donald M. Waller, Kris Verheyen, Lander Baeten, Antoine Becker-Scarpitta, Imre Berki, Markus Bernhardt-Römermann, Jörg Brunet, Hans Van Calster, Markéta Chudomelová, Deborah Closset, Pieter De Frenne, Guillaume Decocq, Frank S. Gilliam, John-Arvid Grytnes, Radim Hédl, Thilo Heinken, Bogdan Jaroszewicz, Martin Kopecký, Jonathan Lenoir, Martin Macek, František Máliš, Tobias Naaf, Anna Orczewska, Petr Petřík, Kamila Reczyńska, Fride Høistad Schei, Wolfgang Schmidt, Alina Stachurska-Swakoń, Tibor Standovár, Krzysztof Świerkosz, Balázs Teleki, Ondřej Vild, Daijiang Li

**Affiliations:** ^1^ Department of Biological Sciences, Louisiana State University, Baton Rouge, LA 70803, USA.; ^2^ Department of Ecology and Evolutionary Biology, University of Arizona, Tucson, AZ 85720, USA.; ^3^ Florida Museum of Natural History, University of Florida, Gainesville, FL 32611, USA.; ^4^ Department of Biological Sciences, Mississippi State University, Mississippi State, MS 39762, USA.; ^5^ Department of Botany, University of Wisconsin-Madison, Madison, WI 53706, USA.; ^6^ Forest and Nature Lab, Department of Environment, Faculty of Bioscience Engineering, Ghent University, B-9000 Melle-Gontrode, Belgium.; ^7^ CIRAD, UMR PVBMT, 97410 Saint Pierre, La Réunion, France.; ^8^ Faculty of Forestry, University of Sopron, Bajcsy Zs. str. 4., H-9400 Sopron, Hungary.; ^9^ Institute of Ecology and Evolution, Friedrich-Schiller-University Jena, Dornburger Str. 159, D-07743 Jena, Germany.; ^10^ German Centre for Integrative Biodiversity Research (iDiv) Halle-Jena-Leipzig, Puschstrasse 4, 04103 Leipzig, Germany.; ^11^ Southern Swedish Forest Research Centre, Swedish University of Agricultural Sciences, Box 190, 23422 Lomma, Sweden.; ^12^ Research Institute for Nature and Forest, Havenlaan 88 bus 73, B-1000 Brussel, Belgium.; ^13^ Institute of Botany, Czech Academy of Sciences, Lidická 25/27, 60200 Brno, Czech Republic.; ^14^ UMR CNRS 7058 “Ecologie et Dynamique des Systèmes Anthropisés” (EDYSAN), Université de Picardie Jules Verne, 1 rue des Louvels, 80000 Amiens, France.; ^15^ Department of Earth and Environmental Sciences, University of West Florida, Pensacola, FL 32514, USA.; ^16^ Department of Biological Sciences, University of Bergen, Postbox 7803, 5020 Bergen, Norway.; ^17^ Department of Botany, Faculty of Science, Palacký University in Olomouc, Šlechtitelů 27, CZ-779 00 Olomouc, Czech Republic.; ^18^ General Botany, Institute of Biochemistry and Biology, University of Potsdam, Maulbeerallee 3, D-14469 Potsdam, Germany.; ^19^ Białowieża Geobotanical Station, Faculty of Biology, University of Warsaw, Sportowa 19, PL-17-230 Warsaw, Poland.; ^20^ Institute of Botany of the Czech Academy of Sciences, Zámek 1, CZ-252 43 Průhonice, Czech Republic.; ^21^ Faculty of Forestry and Wood Sciences, Czech University of Life Sciences Prague, Kamýcká 129, CZ-165 21 Praha 6 - Suchdol, Czech Republic.; ^22^ Technical University in Zvolen, T. G. Masaryka 24, SK-96001 Zvolen, Slovakia.; ^23^ Leibniz Centre for Agricultural Landscape Research (ZALF), Eberswalder Strasse 84, D-15374 Muencheberg, Germany.; ^24^ Institute of Biology Biotechnology and Environmental Protection, Faculty of Natural Sciences, University of Silesia, PL-40-032 Katowice, Poland.; ^25^ Czech University of Life Sciences Prague, Kamýcká 129, CZ-16500 Praha, Suchdol, Czech Republic.; ^26^ Independent Researcher, PL-50-524 Wrocław, Poland.; ^27^ Norwegian Institute of Bioeconomy Research, Thormøhlensgate 55, 5006 Bergen, Norway.; ^28^ Department of Silviculture and Forest Ecology of the Temperate Zones, University of Göttingen, Büsgenweg 1, D-37077 Göttingen, Germany.; ^29^ Institute of Botany, Faculty of Biology, Jagiellonian University in Kraków, Gronostajowa 3, PL-30-387 Kraków, Poland.; ^30^ ELTE Eötvös Loránd University Department of Plant Systematics, Ecology and Theoretical Biology, Institute of Biology, Pázmány P. sétány 1/c, H-1117 Budapest, Hungary.; ^31^ Museum of Natural History, Faculty of Biological Sciences, University of Wrocław, Sienkiewicza 21, PL-50-335 Wrocław, Poland.; ^32^ MTA-DE Lendület Functional and Restoration Ecology Research Group, H-4032 Debrecen, Hungary.; ^33^ Center for Computation & Technology, Louisiana State University, Baton Rouge, LA 70803, USA.

## Abstract

Biological nitrogen fixation is a fundamental part of ecosystem functioning. Anthropogenic nitrogen deposition and climate change may, however, limit the competitive advantage of nitrogen-fixing plants, leading to reduced relative diversity of nitrogen-fixing plants. Yet, assessments of changes of nitrogen-fixing plant long-term community diversity are rare. Here, we examine temporal trends in the diversity of nitrogen-fixing plants and their relationships with anthropogenic nitrogen deposition while accounting for changes in temperature and aridity. We used forest-floor vegetation resurveys of temperate forests in Europe and the United States spanning multiple decades. Nitrogen-fixer richness declined as nitrogen deposition increased over time but did not respond to changes in climate. Phylogenetic diversity also declined, as distinct lineages of N-fixers were lost between surveys, but the “winners” and “losers” among nitrogen-fixing lineages varied among study sites, suggesting that losses are context dependent. Anthropogenic nitrogen deposition reduces nitrogen-fixing plant diversity in ways that may strongly affect natural nitrogen fixation.

## Teaser

Anthropogenic nitrogen deposition reduces nitrogen-fixing plant diversity, but nitrogen-fixer losses are context dependent.

## INTRODUCTION

Bioavailable nitrogen (N) in ecological systems is often limited (*1*–*3*). Nitrogen-fixing plants are able to overcome this limitation, even in nutrient-poor soils, because they form a symbiosis with diazotrophic bacteria that fix N from the atmosphere (*4*, *5*). These N-fixing plants can thus enrich the surrounding soil and modify the available niche space for other organisms, providing valuable ecosystem services for both natural and seminatural systems (*6*, *7*). However, N-fixers and their services are vulnerable to environmental changes, specifically to climate changes and anthropogenic nitrogen depositions (*7*–*12*). Current global temperatures have surpassed 1.1°C over preindustrial levels, global soil moisture has decreased despite overall increases in precipitation, and nitrogen deposition caused by human activities has accelerated since the early 20th century (*13*–*15*) and is unlikely to slow in the near future. Hence, understanding the response of N-fixing symbionts to past environmental changes can help us predict future changes.

Instead of directly investigating the temporal changes in the richness of N-fixing plants in response to environmental change, most studies on N-fixing plant diversity so far have focused on the environmental factors that determine their spatial distribution. This is largely due to the paucity of long-term monitoring datasets on plant community composition. Previous studies identified two main drivers of diversity for N-fixing plants: nutrient availability and climate, especially temperature and aridity (*5*, *7*, *10*–*12*, *16*). Nutrient availability affects the distribution and diversity of N-fixing plants because N-fixing plants differ in their capacity to reduce their investment in root nodules in N-rich environments (*17*–*19*) while symbiosis is costly, putting obligate N-fixing plants at a competitive disadvantage where N inputs are high (*20*). Anthropogenic inputs of N could, therefore, have substantial impacts, affecting N-fixing plants more acutely than co-occurring plants without N-fixing symbiosis. Analyses of N-fixing plant diversity in subtropical and temperate regions also reveal notable effects of temperature, with higher temperatures increasing the abundance of N-fixing trees (*11*). Higher temperatures are associated with higher nodulation and enzymatic activity of the bacterial symbiont (optima between 20° and 37°C) (*21*–*23*). Similar to temperature, the effects of aridity on N-fixing plant diversity have been tested in tropical and temperate ecosystems, with several studies reporting relatively higher N-fixing plant richness and phylogenetic diversity (PD) in more arid environments (*10*, *12*) because high foliar N concentrations, a hallmark of N-fixing plants, increase water-use efficiency (*10*, *24*).

Far less well understood are temporal changes in N-fixing plant diversity and drivers of that change, despite profound human-driven changes to the environment over the past century. For example, anthropogenic activities have markedly increased global temperatures and regional aridity (especially in the Northern Hemisphere) (*14*, *15*), and annual anthropogenic N deposition has more than tripled since the 19th century (*13*). This radical regional shift in N availability may have both direct and indirect impacts on plant diversity through complex interplays with climate change. Such changes are thought to disproportionately affect N-fixing species, decreasing N-fixing plant taxonomic and PD (*8*, *20*) in response to the different ecological needs and sensitivities of N-fixing plant clades (*16*).

**Fig. 1.**
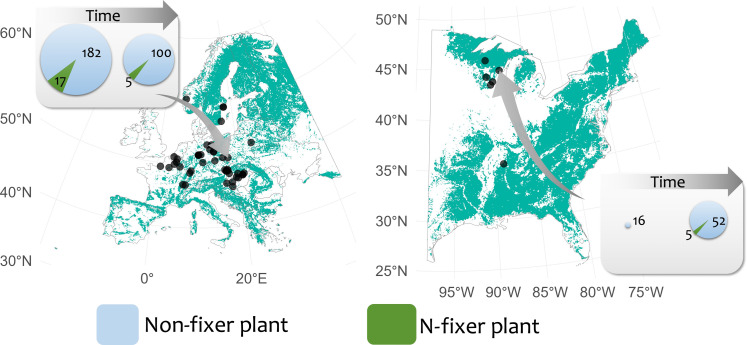
Geographical distribution of the 53 study sites. The sites are located in temperate forests of Europe (*n* = 47) and the US (*n* = 6) and include 971 plots that contain at least one N-fixer species in either one or both of the surveys. Forest cover is shown in green on the map. The two example boxes show the trends for N-fixers (dark green) and non-fixers (light blue) on a site that has lost and a site that has gained both N-fixer and non-fixer species. Pie size is proportional to overall understory richness.

Here, we use long-term vegetation resurvey datasets compiled across temperate forests in Europe and the US to evaluate temporal trends in the diversity of understory forest N-fixing plants and their underlying environmental factors (Fig. 1). In particular, we examine how increases in N deposition, climate warming, and aridity have affected: (i) the proportion of N-fixer species in the community, (ii) PD of N-fixers, and (iii) the proportion of PD of N-fixers in the community. We do so by leveraging the broad temporal and spatial coverage of the forestREplot database version 2.3 (*25*) and using baseline as well as resurveys of 971 vegetation plots. We hypothesize that cumulative N deposition over time has reduced the proportional richness and PD of N-fixers in temperate forests by limiting their competitive advantage over non-fixer species. On the other hand, we expect that past increases in temperature and/or aridity may have resulted in higher proportional richness and PD of N-fixers, given the lower costs of fixing N at higher temperatures and water-use efficiency of N-fixers.

## RESULTS

**Fig. 2.**
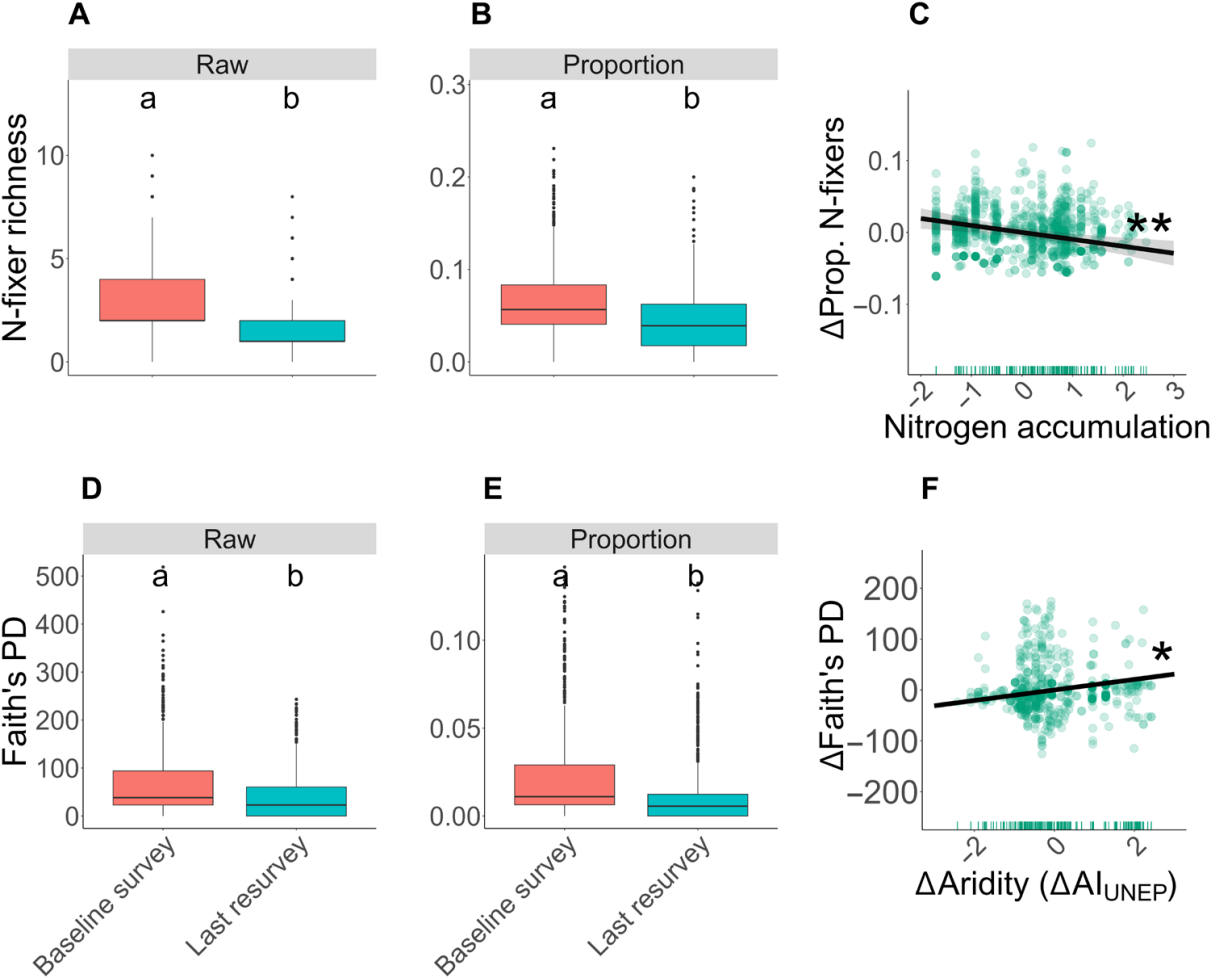
Temporal trends of N-fixer richness and PD. N-fixer richness is expressed in the number of species, whereas N-fixer Faith’s PD is expressed in millions of years. The trends are based on the predicted values from the model formula for each independent variable, by setting all other variables to their mean. The independent variables were standardized to have a mean of 0 and SD of 1.

Plots included, on average, 3 N-fixing plant species with 70 million years of phylogenetic lineages for the baseline survey (N-fixers contributed 6% of the species, 2% of the Faith’s PD) and 1.5 N-fixing plant species with 40 million years of phylogenetic lineages for the last resurvey (N-fixers contributed 4% of the species, 1% of the Faith’s PD). From the baseline survey to the last resurvey, most plots lost N-fixers (65%), losing, on average, 72% of the baseline N-fixer richness. Not only the number but also the proportion of N-fixer species in the community declined, on average, by 2% between the baseline and the last resurvey (Fig. 2). The loss of N-fixer species in comparison to non-fixers was exacerbated by N deposition [maximum likelihood estimation (MLE) of the slope = −0.010, *P* = 0.001; Fig. 2 and table S2), but it did not show any significant response to temporal changes of temperature or aridity (*T*: MLE = 0.001, *P* = 0.668; and AI_UNEP_: MLE = −0.003, *P* = 0.251; fig. S3 and table S2). A deposition of 10 kg of N/ha resulted in an estimated decrease of 1% of N-fixer species proportion in temperate forest communities of Europe and the US (MLE of slope = −0.010, *P* = 0.001; Fig. 2 and table S2). The decrease of N-fixer species proportion was also negatively related to its baseline level (MLE = −0.039, *P* < 0.001; table S2), meaning that the decrease in the proportion of N-fixer species is more acute in communities with higher baseline proportions of N-fixing plants.

Parallel to the decline of the proportion of N-fixer richness, plots lost N-fixer Faith’s PD between surveys (mean = −32.227 millions of years, SD = 77.836; Fig. 2). The increase of aridity alleviated some of the N-fixer Faith’s PD loss (MLE = 10.363, *P* = 0.045; Fig. 2 and table S3). However, the residuals of the raw model were not normally distributed, and the effect of aridity change became marginally significant in the cubic root–transformed model (MLE = 0.504, *P* = 0.056; table S6). This effect was also lost when only including sites with at least two N-fixers in both the baseline and last resurvey (table S3). The change of N-fixer Faith’s PD was negatively related to its baseline level (MLE = −66.778, *P* < 0.001; table S3), meaning that plots with more diverse N-fixer species in the baseline survey lost more PD over time.

The decay of N-fixer Faith’s PD over time was faster than that of the overall community (proportion of N-fixer Faith’s PD versus overall: intercept = −0.009, *P* < 0.001; Fig. 2 and table S4). The proportion of Faith’s PD contained within the N-fixer community declined over time, but it did not respond to any variable other than the baseline proportion of N-fixer Faith’s PD (MLE = −0.024, *P* < 0.001; table S4), so that plots where N-fixer species accounted for a larger proportion of the overall PD in the baseline survey lost, proportionally, a greater amount of N-fixer PD.

**Fig. 3.**
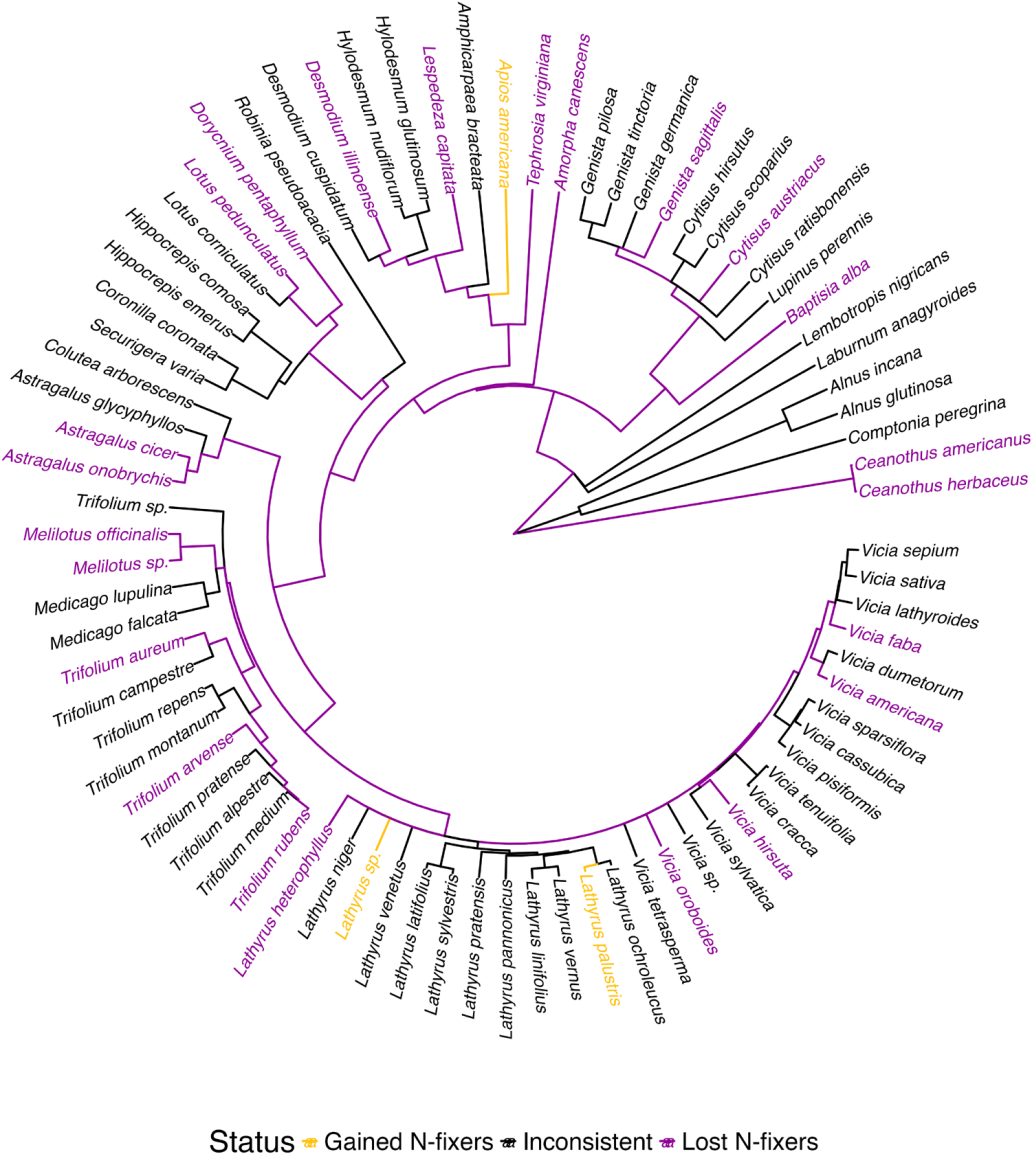
Phylogenetic tree of N-fixer species in the study plots. Species could either appear in both surveys (“conserved,” none), exclusively in the baseline (“lost,” purple), and exclusively in the last resurvey (“gained,” yellow) or display different trends across sites (“inconsistent,” black).

While N-fixer PD decayed over time, N-fixer phylogeny was not a good predictor of the probabilities of N-fixing species being lost or gained, suggesting that no N-fixer clades were consistently lost, conserved, or gained across sites between the baseline surveys and the last resurveys (Pagel’s λ < 0.001, *P* = 1 for the probability of species being lost, gained, and net presence change). At the species level, most species displayed different trends across sites (67.5%, or 54 species) and fewer were either consistently lost (28.8%, or 23 species) or gained (3.8%, or 3 species) (Fig. 3). The haphazard loss of N-fixer species across the phylogenetic tree did not preclude N-fixer guilds (i.e., conserved, lost, and gained species) from displaying different levels of PD (Faith’s PD) (Table 1). In general, sites tended to conserve closely related species of N-fixers and conversely lost the species that contained higher amounts of overall Faith’s PD (Table 1).

**Table 1.** Results from the pairwise comparisons of N-fixer Faith’s PD among conserved, lost, and gained species. These comparisons are based on two linear mixed-effect models with PD as the response, species group as the fixed effect (i.e., lost, gained, or conserved), and site identity as the random intercept. We repeated the models with gained and conserved species as the fixed intercept. The model with conserved species as the intercept shows all comparisons but that between lost and gained species; similarly, the model with gained species as the intercept shows all comparisons but that between conserved and lost species.

Comparison	Value	SE	*t* value	*P* value
	Faith’s PD (df = 769)			
Conserved - Lost	−37.295	5.169	−7.215	<0.001
Conserved - Gained	−23.771	6.435	−3.694	<0.001
Gained - Lost	−13.523	5.405	−2.502	0.013

## DISCUSSION

Temperate forests of Europe and the US are losing species richness and PD of N-fixing plants faster than those of the whole community. The last resurveys across the 971 studied plots (median year = 2012) revealed consistent declines in the proportion of N-fixing plant species richness and PD. Nitrogen deposition was significantly associated with the temporal decline in the proportion of N-fixing plants, whereas changes in temperature and aridity displayed no statistically significant effects on the change in the proportion of N-fixing plants. Loser and winner N-fixing species varied across sites, with no N-fixer clade consistently retained, lost, or gained across sites. The decline of N-fixer PD resulted in the asymmetric loss of phylogenetically distinct N-fixing plant clades, eliminating unique lineages and leaving behind depauperate N-fixing plant communities.

Declines in N-fixing plants and the effect of bioavailable N are consistent with empirical studies (*9*), experiments (*8*), and historical fluctuations of N-fixers in comparison to C:N ratios (*7*). Our results reveal consistent declines of N-fixing plants in temperate forests over the past few decades, raising concerns about a possible future with less N-fixing plant diversity in forests, and possibly less biological N fixation or, at least, less resilient biological N fixation. At the same time, agroecological demands for N-fixing plants will be higher, given higher N mineralization rates as a result of global warming (*7*, *15*) and higher N demands in response to regional increases of aridity among dry systems (given the positive relationship between water-use efficiency and foliar N concentrations) (*7*, *14*).

The lack of significant effects of temperature and aridity may be due not only to the higher sensitivity of N-fixer species to chronically high soil N but also to the relatively slower climate changes in comparison to the rapid accumulation of anthropogenic N. Nitrogen deposition increased almost tenfold between surveys (mean of Δ*N*/*N*_baseline_ = 9.98), whereas temperature and aridity increased more moderately (mean of ΔTemperature/Temperature_baseline_ = 0.14 and mean of ΔPrecipitation/Precipitation_baseline_ = 0.04). The lower temporal change in temperature and aridity contrasts with the broader gradients assessed in past spatial analyses (e.g., ΔAI/AI_min_ ≃ 3 and Δ*T* = 35°C) (*10*, *11*). Alternatively, N-fixing plant communities may experience lags throughout a climatic debt or respond to microclimatic factors (*26*, *27*). We tested the effect of microclimate changes by simultaneously considering the effects of macroclimatic temperature change and canopy cover change (tables S13 to S18). While we did not find any significant correlations, future studies directly measuring microclimatic changes could reveal such trends.

Over the past decades, the decline in the proportion of N-fixing plants in the understory of temperate forests across Europe and the US was accompanied by a decline in PD, which plummeted faster than the PD of the entire plant community. This decline of N-fixer PD may have been attenuated by local increases of aridity or other factors promoting N-fixing strategies (*8*, *10*). In general, N-fixing plant species living in the understory of temperate forests were haphazardly lost from the phylogenetic tree, suggesting that there are either no significant differences in N-fixer resilience to nitrogen deposition among different clades or that the seemingly haphazardous change in N-fixers is mediated by local environmental characteristics and plot historical management (*26*). Although haphazard, plots consistently lost phylogenetically diverse sets of plants, leading to depauperate communities of closely related N-fixing species, consistent with prior studies (*28*).

Few studies have assessed the variability of N-fixing plant species richness susceptibility to N deposition. However, responses to N deposition may be consistent across coexisting N-fixing species (*27*) and vary in response to individual plant and bacteria genotypes as well as local environmental conditions (*29*). Nitrogen deposition reduces the competitive advantage of N-fixing plants and alters their ecological dependencies, modifying N-fixer responses to environmental drivers (e.g., temperature) (*30*), increasing N-fixer susceptibility to inhibitors such as ozone and ultraviolet light (*31*), disrupting plant-mycorrhizal relationships (*32*), and intensifying the competition for other limiting resources (*8*, *33*). The simultaneous context-dependent susceptibility to N deposition with the alteration of the main local ecological drivers and limiting resources is consistent with the selection of guilds of superior competitors, which may outcompete other species depending on the local conditions (e.g., herbivory pressure) and/or priority effects (*8*).

Nitrogen deposition is associated with long-term declines in the proportion of N-fixing plants across temperate forests of Europe and the US. Unexpectedly, changes in temperature and aridity did not contribute to the observed temporal changes in N-fixing diversity, likely reflecting the greater relative importance of N deposition for N-fixing plants. Given the effect of N deposition on N-fixer richness, we should be cautious about predicting future changes in N-fixers based solely on climatic changes without understanding the complex interplays with anthropogenically driven changes in soil nutrient conditions. Declines in N-fixer PD mostly reflect the loss of evolutionarily divergent species, leading to fewer distinct N-fixing lineages. However, no consistent clades of winner or loser species are found, indicating that the response of N-fixing plants to N deposition is driven by local environmental conditions (and possibly priority effects). Therefore, the strategic benefits of temperature and aridity increases for N-fixing species may be curtailed by N deposition, reducing N-fixing richness and their associated ecosystem services.

## MATERIALS AND METHODS

### Plant community data

We used the forestREplot database v.2.3 (*25*) (www.forestreplot.ugent.be) to study temporal changes of nitrogen-fixing plant diversity. We selected 971 pairs of survey/resurvey plots from 53 sites (Fig. 1) that included at least one N-fixing plant in either one or both of the surveys (i.e., the baseline and last resurvey) for the analysis of species richness and two N-fixing plants for those of PD. Baseline surveys for the selected plots were conducted between 1940 and 1999, while last resurveys were conducted between 1995 and 2019 (interval median ± SD, 44 ± 15 years). For plots with multiple resurveys, we only kept the most recent survey in addition to the baseline. We limited our analyses to vascular plant species within the understory layers (1598 species). We assessed whether each plant species was a N-fixer based on the N-fixing symbiotic ability of the genus (*34*, *35*) because many species have not yet undergone definitive assessments of nodule formation and the trait is typically conserved at lower taxonomic levels. This approach has been used widely and has been demonstrated to be sufficient (*12*).

### N-fixer proportion of species richness

For each plot, we calculated the proportion of N-fixers by dividing the richness of N-fixers by the total plant richness in both the baseline survey and the last resurvey. We used the proportion of N-fixer species richness as our dependent variable for conceptual and practical reasons. Conceptually, we aim to assess the temporal dynamics of N-fixer diversity relative to the overall community because general diversity patterns for the study area have been already evaluated (*36*). Practically, using proportions allows us to remove the effect of plot size. In addition, the change of N-fixer richness was significantly correlated with the change in the proportion of N-fixers [coefficient of determination (*R*^2^) = 21%, *P* < 0.001; table S1], suggesting that the trends of changes in proportion of N-fixers observed reflect those changes in N-fixer richness. To assess the effects of N deposition and climate change on the relative diversity of N-fixers, we calculated the difference between the N-fixer proportion in the last resurvey and baseline survey.

### Phylogeny and proportion of N-fixer PD

We computed N-fixer PD for each plot and both surveys (i.e., baseline and resurvey) and the difference between both surveys for each plot because PD change may differ from species richness change (*37*). We derived our plant phylogeny from one of the most complete and updated global phylogenies for seed plants (*38*) and obtained pruned trees for all vascular plants occurring in the vegetation plots using the R package “rtrees” v.1.0.1 in R (*39*). From the 1526 plant species occurring in the selected plots, 1127 plants were already listed in the phylogeny, 354 were added at the genus level, and 45 were added at the family level, including plants that were originally identified at low taxonomic resolutions (i.e., section, genus, or family); PD calculation has been shown to be robust under these conditions (*40*).

We used Faith’s index of PD (Faith’s PD) (*41*) to quantify PD. Faith’s PD quantifies the total branch length in the phylogeny for the species found within a community. In addition, we tested two other metrics of PD: mean pairwise distance (MPD) and mean nearest taxon distance (MNTD) (*42*). We kept the analysis with Faith’s PD in the Results. We included the results for MPD and MNTD in tables S3, S4, S6, and S7 because accurate measurements of MPD and MNTD could only be calculated for a fraction of the plots (i.e., those with at least two N-fixer species on both surveys). We also evaluated the change of N-fixer Faith’s PD in relation to the overall change in Faith’s PD of all plant species. To do so, we computed the proportion of total Faith’s PD contained among N-fixers for both surveys and calculated the difference in N-fixer Faith’s PD proportion between the last resurvey and the baseline survey

$$\begin{array}{c} ∆\frac{\text{Faith}’s\;\text{PD}\;N-\text{fixer}\;}{\text{Faith}’s\;\text{PD}}=\\ \frac{\text{Faith}’s\;\text{PD}\;N-{\text{fixer}}_{\text{re}-\text{survey}}}{\text{Faith}’s\;{\text{PD}}_{\text{re}-\text{survey}}}-\frac{\text{Faith}’s\;\text{PD}\;N-{\text{fixer}}_{\text{baseline}}}{\text{Faith}’s\;{\text{PD}}_{\text{baseline}}} \end{array}$$ (1)

We assessed the change of N-fixer Faith’s PD and proportion of N-fixer Faith’s PD including sites that had at least two species of N-fixers in one of the surveys (i.e., baseline and/or resurvey, 651 plots). We compared these results with the models using only the subset of plots with two or more N-fixers in both surveys (i.e., baseline and resurvey, 194 plots) and reported both in tables S3 and S4. For the models for MPD and MNTD including all 651 plots, we set MPD and MNTD to 0 for surveys with a single N-fixer. We calculated all metrics of PD using R packages “ape” v.5.6.2 (*43*) and “PhyloMeasures” v.2.1 (*44*).

### Nitrogen deposition

We extracted the total N deposition amount in the selected plots from a model providing monthly data of N deposition rate (grams of N per square meter per year) from 1860 to 2016 at a spatial resolution of 0.5° (about 50 km at the equator) (*13*). We summed the monthly N deposition rates of each year to obtain a time series of annual cumulated N deposition rates. Some of the resurveys were conducted after 2016; in those cases, we used the N deposition rates from 2016, assuming that N deposition did not change notably. Because of the coarse spatial resolution and because bioavailable N is the variable of interest, we calculated the cumulative total N deposition between the two survey years for each site by adding the annual N deposition amount of all the years within the time interval. We then used the same value of N deposition for all plots within each site.

### Temperature

We compiled temperature data from the Climate Research Unit v.4.0.7 (*45*), which provides global monthly temperature averages from 1901 to 2022 at a 0.5° spatial resolution. We extracted the temperature using the geographical coordinates of our study plots and averaged the monthly temperature data into annual means using the R package “terra” v.1.7.17 (*46*). We then used moving averages to compute the mean annual temperature over a 5-year period including the year of the survey (i.e., baseline survey or last resurvey) and its preceding 4 years. We evaluated temperature change by computing the difference in mean annual temperature between the two periods of 5 years preceding the baseline and last resurvey.

### Aridity

We obtained annual aridity data from 1901 to 2019 at a 0.5° resolution (*14*) and used the measurements of aridity based on the United Nations Environment Programme (UNEP) aridity index (*47*)

$${\text{AI}}_{\text{UNEP}}=\frac{P}{\text{PET}}$$ (2)

where *P* is the annual precipitation and PET the potential evapotranspiration. The AI_UNEP_ compares precipitation with evapotranspiration, taking higher values for lower aridity. We extracted the annual evapotranspiration at the center of our study plots and used moving means to calculate the mean annual aridity index over a 5-year period, including the year of the survey and its preceding 4 years using the R package terra v.1.7.23 (*46*). We calculated the change in the aridity index as the difference in aridity between the periods of 5 years for the last resurvey and baseline survey.

### Statistical analysis

We performed linear mixed-effects models with the change of N-fixer proportion, Faith’s PD, or proportion of N-fixer Faith’s PD as the response variable; N accumulation, changes in temperature, changes in aridity, and the baseline of the response variable (e.g., Faith’s PD at the baseline survey) as the fixed effects; and the identity of the study site (dataset) as the random intercept using nlme v.3.1.157 in R (*48*). We included the baseline response variable to account for the potential effect of regression to the mean (*49*). The results for the models using the subset of 194 plots revealed no consistent significant effects, probably because of the much smaller sample size. We discuss the models with the full dataset in the Results and Discussion but include both models in tables S3 and S4. We standardized the independent variables to have mean of 0 and SD of 1, checked for outliers and influential points, corroborated the parametric conditions (i.e., normality of residuals, homoscedasticity, and linearity), and verified that the fixed variables were not collinear using the “vif” function in the R package “car” v.3.1.1 (*50*).

We repeated the analyses controlling for the effects of taxonomic resolution and canopy cover because plant responses to global warming in our study sites are modulated by changes in the canopy cover (*51*). To control for taxonomic resolution, we removed 4782 instances where specimens were identified to morphospecies, genus, or higher taxonomic levels (e.g., *Lathyrus* sp. in Fig. 3) and repeated the models. We maintained the low-resolution IDs for the main analyses because most cases involved species identified to genera in surveys without any congeneric species. To assess the effect of canopy cover, we repeated the models by adding the fixed effect of the change of canopy cover. We found only slight differences between the unadjusted models and those controlling for taxonomic accuracy and canopy cover. We discuss the raw models in the Results and Discussion and present the modified models in tables S8 to S18. We also repeated the analyses using vegetation abundance data (with percentage of vegetative cover within a plot as a proxy) and evaluating both the overall change of N-fixer abundance and the change in the proportion of abundance contributed by N-fixer species. Cover data were collected for 841 plots by multiple observers and across years. Plant cover data were not standardized across samples, and, thus, these results should be taken with some caution. We present the models for N-fixer abundance in tables S19 to S22.

We evaluated the PD of N-fixing species that were either lost, gained, or conserved among surveys. We define lost species as those present on a plot’s baseline survey but absent on its last resurvey, gained species as those present in the last resurvey but absent in the baseline survey, and conserved species as those present in both surveys. First, we assessed whether the probability of a species being lost or gained is phylogenetically conserved. We used the proportion of plots in which a species was lost or gained as a measure of the probability of being, respectively, lost or gained. We also computed the net change

$$∆P_{i}=\frac{\;{PG}_{i}-{PL}_{i}}{{PC}_{i}+PG_{i}+{PL}_{i}}$$ (3)

where *i* refers to the target species, Δ*P* refers to the relative change in the number of plots where the species is present, *P*G refers to the number of plots where the species was gained, *P*L refers to the number of plots where the species was lost, and *P*C refers to the number of plots where the species was conserved. We calculated Pagel’s λ (*52*) for the probability of being lost, gained, and the net change using the R package “phytools” v.1.9.16 (*53*).

We also compared the PD contained among species that have been lost, gained, and conserved on each plot. First, we separated the observed plants of each plot in three categories: lost (only observed in the baseline survey), gained (only observed in the last resurvey), and conserved (observed in both surveys). We then calculated the Faith’s PD, MPD, and MNTD of each group of species (i.e., gained, lost, and conserved) across all sites. We compared the PD across the three groups using linear-mixed effects models with the index of PD as the response variable (i.e., Faith’s PD, MPD, or MNTD), the group classification as the fixed effect (i.e., gained N-fixers, lost, and conserved), and site identity as the random intercept. We computed multiple models to obtain the results for all possible pairwise comparisons. We present the pairwise comparisons for Faith’s PD obtained from the models in the Results. We present the results for MPD and MNTD in the tables S3, S4, S6, and S7.
