# Supplementary material for: Long-term nitrogen deposition reduces the diversity of nitrogen-fixing plants
Source: Sci Adv. 2024 Oct 18;10(42):eadp7953. doi: 10.1126/sciadv.adp7953 (PMC11488573; doi:10.1126/sciadv.adp7953)
Supplement: Supplementary file 1 — Supplementary Text Figs. S1 to S3 Tables S1 to S22 [file sciadv.adp7953_sm.pdf]

Science Advances
AAAS

Supplementary Materials for

Long-term nitrogen deposition reduces the diversity of nitrogen-fixing plants

Pablo Moreno-García *et al.*

Corresponding author: Pablo Moreno-García, pablo.paul.paolo@gmail.com; Daijiang Li, daijianglee@gmail.com

Sci. Adv. **10**, eadp7953 (2024)
DOI: 10.1126/sciadv.adp7953

This PDF file includes:

Supplementary Text
Figs. S1 to S3
Tables S1 to S22

Supplementary Text

N-fixer proportion vs. richness

In the main text, we evaluated the effects of N deposition and climate change on the proportion of N-fixer species, rather than on N-fixer richness. By using the proportion, we were able to compare the trends among sites that differ widely in their extension, as it often happens across the forestREplot network. However, temporal changes in the proportion of N-fixers may be explained not only by decreases in N-fixer richness, but also by increases of non-fixer richness relative to N-fixers. Thus, we evaluated the relationship between the temporal change in N-fixer richness and the proportion of N-fixers in the community. We found that the change in N-fixer richness and the proportion of N-fixers are highly correlated (MLE = 0.008, P-value <0.001), and that the change in N-fixer richness explains a sizable amount of the variance of N-fixer proportion (adjusted R^2 = 20.8%) (Fig. S1, Table S1). Given the high correlation among these variables and the advantages of using the change of the proportion of N-fixers to draw comparisons among our sites, we decided to use the proportion of N-fixers on our analyses.

LME model effects tables

We include the effects tables for the LME models presented in the main text, as well as the models for all metrics of phylogenetic diversity (Faith's PD, MPD, and MNTD), including the whole plot set and only the subset of plots with ≥2 N-fixer species in both surveys (Tables S2 - S5).

LME cubic-root transformed model effects table

Some models had issues with the normality of the residuals. We repeated the models using a cubic-root transformation (Tables S6, S7).

Change of richness among surveys at the site level

We include a map depicting changes on plant richness at the site level (multiple plots per site) for nitrogen-fixing and non-fixing plant species (Fig. S2).

Model effects tables with taxa ID-ed at species resolution

We repeated the models excluding the records identified at lower resolutions (taxa not identified to species) (Tables S8 - S12).

Canopy cover models

We repeated the models including the effect of canopy cover, to account for microclimate changes as the combination between air temperature and canopy cover (Tables S13 - S18). In the model including changes in canopy cover the relationship between N-fixer proportion in the community and nitrogen accumulation was lost. However, this is not due to the inclusion of canopy cover in the model, but by the selection of the subset of sites that included measurements of plant abundances, as it can be seen by the results using the same model without the effect of the change in canopy cover.

Species abundance models

We repeated the models using instead the change of N-fixer abundance between surveys (using percentage of vegetative cover in a plot as a proxy for abundance), as well as the change in the proportion of overall abundance contained among N-fixing species (Tables S19–S22). It is important to note that plant cover estimates were conducted by many observers, who did not standardize their observations together. Therefore, the results obtained from these analyses should be taken with some caution.

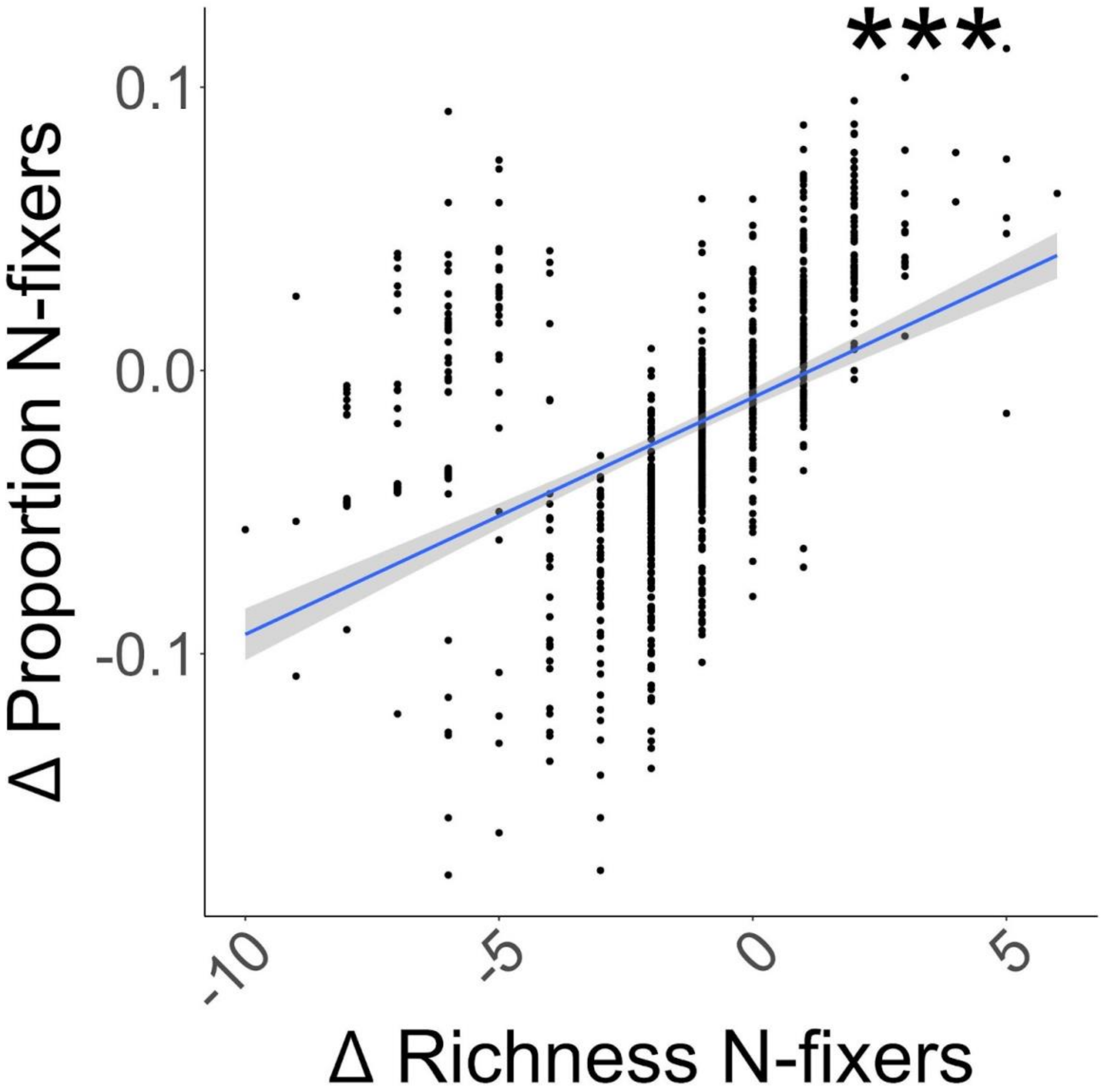

Fig. S1.

Relationship between the change in N-fixer richness and the change of the proportion of forest N-fixers.

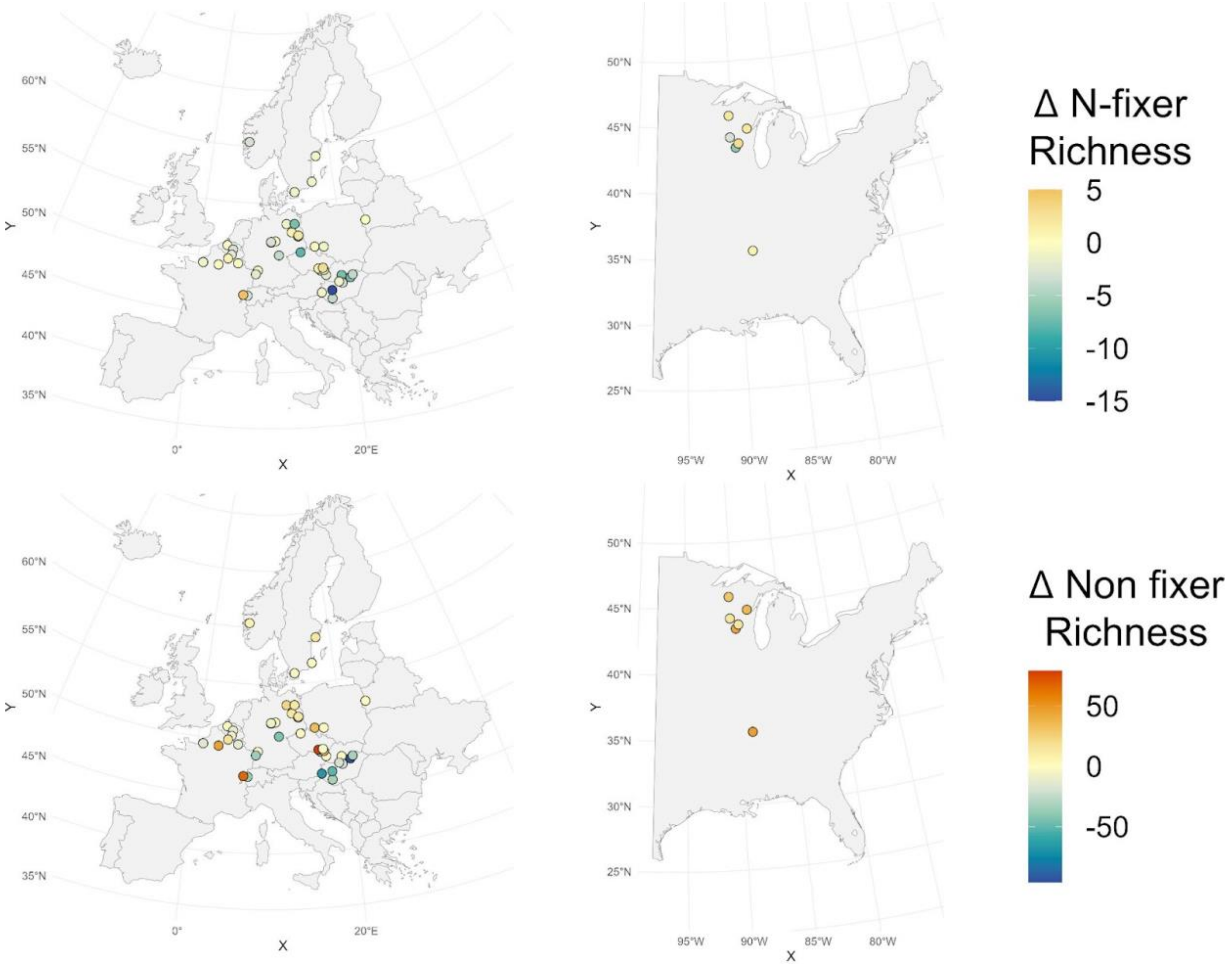

Fig. S2.

Change of species richness from the baseline survey to the last resurvey (in absolute numbers). The maps are based on the selected 53 forestREplot sites, which include 971 plots in European and USA temperate forests. The upper panel portrays changes of N-fixer plant richness, while the panel below reveals changes of non-fixer plant richness. While our analyses are based on changes at the plot level, this figure portrays changes at each site (i.e., containing multiple plots). To assess site data, we pooled the species from the plots for each site and assessed the overall change among surveys.

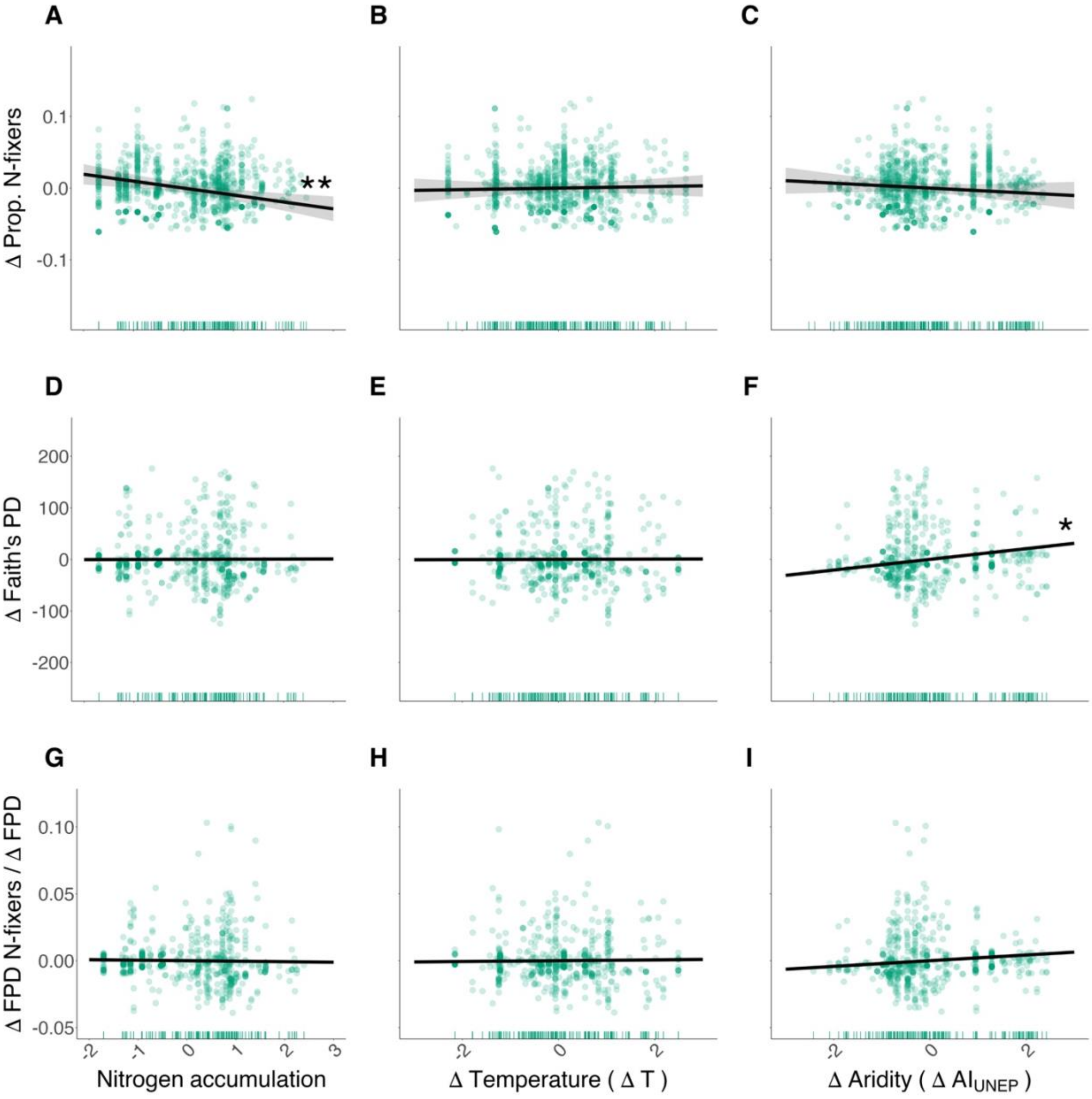

Fig. S3.

Trends of N-fixer richness and PD. N-fixer richness is expressed in the number of species, whereas N-fixer Faith's PD is expressed in millions of years. The trends are based on predicted values from the model formula for each independent variable, by setting all other variables to their mean. The independent variables were standardized to have a mean of 0 and standard deviation of 1.

Table S1.

Summary table of the relationship between the change in N-fixer richness and the change of the proportion of forest N-fixers.

Variable	MLE	Std. Error	P-value
**Intercept**	*-0.010*	*0.002*	*<0.001*
**Δ N-fixer richness**	*0.008*	*0.001*	*<0.001*
**Multiple R^2**	0.209		
**Adjusted R^2**	0.208		

Table S2.

Predicted effects of nitrogen deposition, temperature, and aridity on the proportion of forest N-fixers. The full model includes the site as a random effect for intercept.

Variable	MLE	Std. Error	P-value
**Intercept**	*-0.019*	*0.003*	*<0.001*
**Nitrogen deposition**	*-0.010*	*0.003*	*0.001*
**Δ Temperature**	0.001	0.002	0.668
**Δ Aridity**	-0.003	0.003	0.251
**Baseline N-fixer proportion**	*-0.039*	*0.001*	*<0.001*
**Marginal R^2**	0.570		
**Conditional R^2**	0.688		

Table S3.

Predicted effects of nitrogen deposition, temperature, and aridity on N-fixer PD. The full models include the site as a random effect for intercept. When we limited the analysis to only have plots that have at least 2 N-fixers from both surveys, no predictors were significant, probably because of the much smaller sample size. We set MPD and MNTD of the surveys with 1 N-fixer to 0. The model of MNTD for sites with 2 or more N-fixers in both surveys excludes 1 outlier.

	All surveys			Surveys with ≥ 2 N-fixers		
**Variable**	**MLE**	**Std. Error**	**P-value**	**MLE**	**Std. Error**	**P-value**
**Faith's PD (df = 603)**				**(df = 165)**		
**Intercept**	*-28.511*	*4.853*	*<0.001*	*-34.676*	*8.781*	*<0.001*
**Nitrogen deposition**	*0.278*	*5.145*	*0.957*	13.866	9.247	0.136
**Δ Temperature**	0.260	4.265	0.951	8.552	7.290	0.242
**Δ Aridity**	*10.363*	*5.162*	*0.045*	7.983	10.517	0.449
**Baseline Faith's PD**	-66.778	2.248	*<0.001*	*-75.614*	*4.117*	*<0.001*
**Marginal R^2**	0.606			0.609		
**Conditional R^2**	0.696			0.731		
**MPD (df = 602)**				**(df = 165)**		
**Intercept**	*-11.324*	*3.468*	*<0.001*	-4.844	4.390	0.271
**Nitrogen deposition**	0.267	3.657	0.942	9.152	4.701	0.053
**Δ Temperature**	-0.672	3.005	0.823	3.482	3.778	0.358
**Δ Aridity**	*8.214*	*3.657*	*0.025*	1.377	5.312	0.796
**Baseline MPD**	*-38.751*	*1.552*	*<0.001*	-33.992	2.417	*<0.001*
**Marginal R^2**	0.527			0.503		
**Conditional R^2**	0.644			0.631		
**MNTD (df = 602)**				**(df = 164)**		
**Intercept**	*-6.633*	*3.177*	*0.037*	-0.193	3.198	0.952
**Nitrogen deposition**	1.052	3.346	0.753	1.345	2.925	0.646
**Δ Temperature**	-1.374	2.740	0.616	-1.537	3.918	0.695
**Δ Aridity**	*7.817*	*3.338*	*0.020*	6.885	3.534	0.053
**Baseline MNTD**	*-35.767*	*1.385*	*<0.001*	*-26.835*	*2.077*	*<0.001*
**Marginal R^2**	0.536			0.481		
**Conditional R^2**	0.654			0.567		

Table S4.

Predicted effects of nitrogen deposition, temperature, and aridity on proportion of forest N-fixer PD and overall forest PD. The full models include site as a random effect for intercept. We set MPD and MNTD of the surveys with 1 N-fixer to 0. The models of Faith's PD and MNTD for sites with 2 or more N-fixers in both surveys exclude, respectively, 4 and 1 outliers.

	All surveys			Surveys with ≥ 2 N-fixers		
**Variable**	**MLE**	**Std. Error**	**P-value**	**MLE**	**Std. Error**	**P-value**
**Faith's PD (df = 602)**				**(df = 162)**		
**Intercept**	*-0.009*	*0.002*	*<0.001*	*-0.012*	*0.003*	*<0.001*
**Nitrogen deposition**	0.000	0.002	0.832	0.004	0.003	0.180
**ΔTemperature**	0.000	0.001	0.834	0.002	0.002	0.409
**Δ Aridity**	0.002	0.002	0.223	0.002	0.003	0.515
**Baseline Faith's PD**	*-0.024*	*0.001*	*<0.001*	*-0.028*	*0.001*	*<0.001*
**Marginal R^2**	0.616			0.680		
**Conditional R^2**	0.686			0.784		
**MPD (df = 602)**				**(df = 165)**		
**Intercept**	*-0.047*	*0.013*	*<0.001*	-0.014	0.018	0.442
**Nitrogen deposition**	-0.001	0.014	0.962	*0.041*	*0.019*	*0.036*
**Δ Temperature**	0.001	0.011	0.953	0.016	0.015	0.314
**Δ Aridity**	0.025	0.014	0.072	0.002	0.022	0.934
**Baseline MPD**	*-0.151*	*0.006*	*<0.001*	-0.137	0.010	*<0.001*
**Marginal R^2**	0.538			0.490		
**Conditional R^2**	0.648			0.623		
**MNTD (df = 602)**				**(df = 164)**		
**Intercept**	*-0.076*	*0.031*	*0.014*	-0.015	0.036	0.670
**Nitrogen deposition**	0.008	0.032	0.794	*0.091*	*0.039*	*0.021*
**Δ Temperature**	-0.023	0.027	0.377	-0.006	0.032	0.840
**Δ Aridity**	0.057	0.032	0.078	-0.009	0.044	0.833
**Baseline MNTD**	*-0.310*	*0.013*	*<0.001*	*-0.239*	*0.020*	*<0.001*
**Marginal R^2**	0.486			0.417		
**Conditional R^2**	0.621			0.548		

Table S5.

Results from the pairwise comparisons of N-fixer MPD and MNTD among conserved, lost, and gained species (between the baseline and last resurvey). These comparisons are based on two linear mixed-effect models with phylogenetic diversity as the response, species group as the fixed effect (i.e., lost, gained, or conserved) and the random intercept of site identity. We repeated the models with either conserved or gained species as reference category to assess all pairwise comparisons.

Comparison	Value	Std. Error	t-value	P-value
MPD (df = 769)				
Conserved - Lost	*-14.986*	*3.296*	*-4.546*	*<0.001*
Conserved - Gained	*-14.614*	*4.109*	*-3.556*	*<0.001*
Gained - Lost	-0.372	3.451	-0.108	0.914
MNTD (df = 769)				
Conserved - Lost	*-8.603*	*3.178*	*-2.707*	*0.007*
Conserved - Gained	*-12.009*	*3.963*	*-3.031*	*0.003*
Gained - Lost	3.406	3.328	1.023	0.306

Table S6.

Predicted effects of nitrogen deposition, temperature, and aridity on cubic-root transformed N-fixer PD. The full models include site as a random effect for intercept. We set MPD and MNTD of the surveys with 1 N-fixer to 0. The model of Faith's PD with multiple N-fixers on both baseline survey and last resurvey did not need to be transformed.

	All surveys		
**Variable**	**MLE**	**Std. Error**	**P-value**
**Faith's PD (df = 602)**			
**Intercept**	*-1.272*	*0.249*	*<0.001*
**Nitrogen deposition**	-0.328	0.261	0.209
**ΔTemperature**	0.054	0.217	0.802
**Δ Aridity**	0.504	0.263	0.056
**Baseline Faith's PD**	*-2.180*	*0.112*	*<0.001*
**Marginal R^2**	0.422		
**Conditional R^2**	0.563		
**MPD (df = 602)**			
**Intercept**	*-0.738*	*0.237*	*0.002*
**Nitrogen deposition**	*0.005*	*0.248*	*0.985*
**Δ Temperature**	-0.130	0.204	0.524
**Δ Aridity**	*0.576*	*0.248*	*0.021*
**Baseline MPD**	*-1.859*	*0.104*	*<0.001*
**Marginal R^2**	0.376		
**Conditional R^2**	0.539		
**MNTD (df = 602)**			
**Intercept**	*-1.115*	*0.270*	*<0.001*
**Nitrogen deposition**	-0.020	0.286	0.945
**Δ Temperature**	-0.089	0.234	0.704
**Δ Aridity**	*0.586*	*0.285*	*0.040*
**Baseline MNTD**	*-1.832*	*0.120*	*<0.001*
**Marginal R^2**	0.309		
**Conditional R^2**	0.497		

Table S7.

Predicted effects of nitrogen deposition, temperature, and aridity on cubic-root transformed ratio of forest N-fixer PD and overall forest PD. The full models include site as a random effect for intercept. We set MPD and MNTD of the surveys with 1 N-fixer to 0. The model of Faith's PD for all surveys excludes 1 outlier. The model of Faith's PD with multiple N-fixers on both baseline survey and last resurvey did not need to be transformed.

	All surveys		
**Variable**	**MLE**	**Std. Error**	**P-value**
**Faith's PD (df = 601)**			
**Intercept**	*-0.083*	*0.017*	*<0.001*
**Nitrogen deposition**	-0.022	0.018	0.240
**Δ Temperature**	0.010	0.015	0.515
**Δ Aridity**	0.024	0.018	0.185
**Baseline Faith's PD**	*-0.158*	*0.008*	*<0.001*
**Marginal R^2**	0.433		
**Conditional R^2**	0.556		
**MPD (df = 602)**			
**Intercept**	*-0.119*	*0.036*	*<0.001*
**Nitrogen deposition**	-0.005	0.039	0.904
**Δ Temperature**	-0.004	0.032	0.888
**Δ Aridity**	*0.078*	*0.038*	*0.043*
**Baseline MPD**	*-0.289*	*0.017*	*<0.001*
**Marginal R^2**	0.369		
**Conditional R^2**	0.516		
**MNTD (df = 602)**			
**Intercept**	*-0.140*	*0.049*	*0.004*
**Nitrogen deposition**	0.017	0.052	0.739
**Δ Temperature**	-0.058	0.042	0.169
**Δ Aridity**	*0.109*	*0.051*	*0.034*
**Baseline MNTD**	*-0.361*	*0.022*	*<0.001*
**Marginal R^2**	0.347		
**Conditional R^2**	0.504		

Table S8.

Predicted effects of nitrogen deposition, temperature, and aridity on the proportion of forest N-fixers. The full model includes the site as a random effect for intercept. This model excludes 1 outlier.

Variable	MLE	Std. Error	P-value
**Intercept**	*-0.021*	*0.003*	*<0.001*
**Nitrogen deposition**	*-0.010*	*0.003*	*0.001*
**Δ Temperature**	0.001	0.003	0.639
**Δ Aridity**	-0.004	0.003	0.256
**Baseline N-fixer proportion**	*-0.041*	*0.001*	*<0.001*
**Marginal R^2**	0.576		
**Conditional R^2**	0.688		

Table S9.

Predicted effects of nitrogen deposition, temperature, and aridity on N-fixer PD. The full models include site as a random effect for intercept. We set MPD and MNTD of the surveys with 1 N-fixer to 0. We excluded 1 outlier for the Faith's PD (for the survey with at least 2 N-fixers in both surveys).

	All surveys			Surveys with ≥ 2 N-fixers		
**Variable**	**MLE**	**Std. Error**	**P-value**	**MLE**	**Std. Error**	**P-value**
**Faith's PD (df = 597)**				**(df = 162)**		
**Intercept**	*-28.238*	*4.888*	*<0.001*	*-40.622*	*7.770*	*<0.001*
**Nitrogen deposition**	1.519	5.163	0.769	10.145	8.447	0.231
**Δ Temperature**	-1.675	4.286	0.696	6.647	7.007	0.344
**Δ Aridity**	*10.390*	*5.192*	*0.046*	-0.405	9.498	0.966
**Baseline Faith's PD**	-66.607	2.249	*<0.001*	-77.535	4.492	*<0.001*
**Marginal R^2**	0.606			0.615		
**Conditional R^2**	0.697			0.690		
**MPD (df = 597)**				**(df = 162)**		
**Intercept**	*-11.353*	*3.507*	*0.001*	-7.882	4.086	0.055
**Nitrogen deposition**	0.741	3.691	0.841	6.315	4.652	0.176
**Δ Temperature**	-1.399	3.034	0.645	3.326	3.873	0.392
**Δ Aridity**	*8.280*	*3.697*	*0.025*	-1.897	5.037	0.707
**Baseline MPD**	-39.007	1.562	*<0.001*	*-33.186*	*2.963*	*<0.001*
**Marginal R^2**	0.528			0.426		
**Conditional R^2**	0.646			0.499		
**MNTD (df = 597)**				**(df = 162)**		
**Intercept**	*-6.752*	*3.207*	*0.036*	-1.006	3.465	0.772
**Nitrogen deposition**	*1.483*	*3.378*	*0.661*	5.581	4.057	0.171
**Δ Temperature**	-1.738	2.768	0.530	1.847	3.367	0.584
**Δ Aridity**	*8.295*	*3.372*	*0.014*	-2.527	4.278	0.556
**Baseline MNTD**	*-36.205*	*1.396*	*<0.001*	*-28.127*	*2.741*	*<0.001*
**Marginal R^2**	0.540			0.388		
**Conditional R^2**	0.657			0.447		

Table S10.

Predicted effects of nitrogen deposition, temperature, and aridity on proportion of forest N-fixer PD and overall forest PD. The full models include site as a random effect for intercept. We set MPD and MNTD of the surveys with 1 N-fixer to 0.

	All surveys			Surveys with ≥ 2 N-fixers		
**Variable**	**MLE**	**Std. Error**	**P-value**	**MLE**	**Std. Error**	**P-value**
**Faith's PD (df = 597)**				**(df = 162)**		
**Intercept**	*-0.010*	*0.002*	*<0.001*	*-0.011*	*0.003*	*0.001*
**Nitrogen deposition**	0.000	0.002	0.962	0.005	0.004	0.155
**Δ Temperature**	-0.001	0.002	0.723	0.002	0.003	0.544
**Δ Aridity**	0.002	0.002	0.266	0.000	0.004	0.914
**Baseline Faith's PD**	*-0.026*	*0.001*	*<0.001*	-0.030	0.002	*<0.001*
**Marginal R^2**	0.637			0.608		
**Conditional R^2**	0.708			0.715		
**MPD (df = 597)**				**(df = 162)**		
**Intercept**	*-0.047*	*0.013*	*<0.001*	-0.030	0.016	0.057
**Nitrogen deposition**	0.001	0.014	0.925	-0.029	0.018	0.108
**Δ Temperature**	-0.002	0.011	0.832	0.016	0.015	0.287
**Δ Aridity**	0.025	0.014	0.075	-0.011	0.019	0.583
**Baseline MPD**	*-0.152*	*0.006*	*<0.001*	*-0.136*	*0.012*	*<0.001*
**Marginal R^2**	0.541			0.432		
**Conditional R^2**	0.562			0.497		
**MNTD (df = 597)**				**(df = 162)**		
**Intercept**	*-0.079*	*0.031*	*0.012*	-0.025	0.039	0.517
**Nitrogen deposition**	0.017	0.032	0.597	0.073	0.044	0.096
**Δ Temperature**	-0.028	0.027	0.295	*-0.020*	*0.036*	*0.576*
**Δ Aridity**	0.064	0.032	0.050	*-0.027*	*0.048*	*0.576*
**Baseline MNTD**	*-0.319*	*0.013*	*<0.001*	*-0.275*	*0.027*	*<0.001*
**Marginal R^2**	0.499			0.384		
**Conditional R^2**	0.632			0.471		

Table S11.

Predicted effects of nitrogen deposition, temperature, and aridity on cubic-root transformed N-fixer PD. The full models include site as a random effect for intercept. We set MPD and MNTD of the surveys with 1 N-fixer to 0. The model of Faith's PD with multiple N-fixers on both baseline survey and last resurvey did not need to be transformed.

	All surveys			Surveys with ≥ 2 N-fixers		
**Variable**	**MLE**	**Std. Error**	**P-value**	**MLE**	**Std. Error**	**P-value**
**Faith's PD (df = 597)**						
**Intercept**	*-1.296*	*0.249*	*<0.001*			
**Nitrogen deposition**	*-0.307*	*0.261*	*0.240*			
**Δ Temperature**	0.036	0.217	0.868			
**Δ Aridity**	0.462	0.263	0.080			
**Baseline Faith's PD**	-2.173	0.113	*<0.001*			
**Marginal R^2**	0.418					
**Conditional R^2**	0.558					
**MPD (df = 597)**				**(df = 162)**		
**Intercept**	*-0.746*	*0.241*	*0.002*	-0.454	0.375	0.228
**Nitrogen deposition**	0.030	0.250	0.905	0.488	0.401	0.225
**Δ Temperature**	-0.177	0.206	0.392	0.021	0.317	0.947
**Δ Aridity**	*0.605*	*0.251*	*0.016*	0.073	0.450	0.872
**Baseline MPD**	*-1.887*	*0.104*	*<0.001*	*-2.051*	*0.201*	*<0.001*
**Marginal R^2**	0.384			0.357		
**Conditional R^2**	0.549			0.538		
**MNTD (df = 597)**				**(df = 162)**		
**Intercept**	*-0.566*	*0.213*	*0.008*	-0.129	0.241	0.592
**Nitrogen deposition**	*0.073*	*0.236*	*0.757*	0.279	0.290	0.337
**Δ Temperature**	-0.135	0.193	0.484	0.104	0.245	0.673
**Δ Aridity**	*0.636*	*0.233*	*0.006*	-0.276	0.298	0.355
**Baseline MNTD**	*-1.699*	*0.106*	*<0.001*	*-1.647*	*0.213*	*<0.001*
**Marginal R^2**	0.340			0.271		
**Conditional R^2**	0.462			0.315		

Table S12.

Predicted effects of nitrogen deposition, temperature, and aridity on cubic-root transformed ratio of forest N-fixer PD and overall forest PD. The full models include site as a random effect for intercept. We set MPD and MNTD of the surveys with 1 N-fixer to 0. The model for MPD with multiple N-fixers on both baseline survey and last resurvey did not need to be transformed.

	All surveys			Surveys with ≥ 2 N-fixers		
**Variable**	**MLE**	**Std. Error**	**P-value**	**MLE**	**Std. Error**	**P-value**
**Faith's PD (df = 597)**						
**Intercept**	*-0.084*	*0.017*	*<0.001*	*-0.084*	*0.031*	*0.007*
**Nitrogen deposition**	-0.014	0.019	0.456	0.018	0.032	0.571
**Δ Temperature**	0.000	0.015	0.983	0.010	0.026	0.710
**Δ Aridity**	0.026	0.019	0.170	-0.009	0.037	0.812
**Baseline Faith's PD**	*-0.156*	*0.008*	*<0.001*	*-0.198*	*0.016*	*<0.001*
**Marginal R^2**	0.416			0.462		
**Conditional R^2**	0.537			0.624		
**MPD (df = 597)**						
**Intercept**	*-0.119*	*0.037*	*0.001*			
**Nitrogen deposition**	-0.002	0.039	0.964			
**Δ Temperature**	-0.020	0.032	0.535			
**Δ Aridity**	*0.083*	*0.039*	*0.035*			
**Baseline MPD**	*-0.294*	*0.017*	*<0.001*			
**Marginal R^2**	0.384					
**Conditional R^2**	0.535					
**MNTD (df = 597)**				**(df = 162)**		
**Intercept**	*-0.141*	*0.050*	*0.005*	-0.021	0.081	0.794
**Nitrogen deposition**	0.023	0.052	0.659	*0.197*	*0.087*	*0.025*
**Δ Temperature**	-0.078	0.043	0.071	*-0.100*	*0.069*	*0.149*
**Δ Aridity**	*0.110*	*0.052*	*0.036*	0.033	0.098	0.737
**Baseline MNTD**	*-0.374*	*0.022*	*<0.001*	-0.392	0.044	*<0.001*
**Marginal R^2**	0.367			0.324		
**Conditional R^2**	0.526			0.504		

Table S13.

Predicted effects of baseline conditions and changes of nitrogen deposition, temperature, aridity, and canopy cover on the proportion of forest N-fixers (Marginal R^2 = 0.563, Conditional R^2 = 0.627). The full model includes site as a random effect for intercept. The model on the right excludes the effect of canopy cover.

Variable	MLE	Std. Error	P-value	MLE	Std. Error	P-value
**Intercept**	*-0.011*	*0.003*	*<0.001*	*-0.011*	*0.003*	*<0.001*
**Nitrogen accumulation**	0.000	0.003	0.993	0.000	0.003	0.993
**Δ Temperature**	0.001	0.002	0.744	0.001	0.002	0.744
**Δ Aridity**	0.007	0.004	0.100	0.007	0.004	0.099
**Δ Cover**	0.000	0.001	>0.999			
**Baseline N-fixer prop**	*-0.032*	*0.002*	*<0.001*	*-0.032*	*0.001*	*<0.001*
**Marginal R^2**	0.563			0.564		
**Conditional R^2**	0.627			0.628		

Table S14.

Predicted effects of baseline conditions and changes of nitrogen deposition, temperature, and aridity on the cubic root-transformed proportion of forest N-fixers (Marginal $R^2 = 0.359$, Conditional $R^2 = 0.432$). The model includes the site as a random effect for intercept.

Variable	MLE	Std. Error	P-value	MLE	Std. Error	P-value
**Intercept**	*-0.094*	*0.024*	*<0.001*	*-0.094*	*0.024*	*<0.001*
**Nitrogen accumulation**	-0.010	0.024	0.678	-0.010	0.024	0.677
**Δ Temperature**	0.008	0.018	0.671	0.007	0.018	0.673
**Δ Aridity**	0.058	0.032	0.071	0.058	0.032	0.067
**Δ Cover**	0.003	0.011	0.808			
**Baseline N-fixer proportion**	*-0.165*	*0.012*	*<0.001*	*-0.166*	*0.012*	*<0.001*
**Marginal R^2**	0.359			0.359		
**Conditional R^2**	0.432			0.432		

Table S15.

Predicted effects of baseline conditions and changes of nitrogen deposition, temperature, and aridity on N-fixer PD. The full model includes the site as a random effect for intercept. We set MPD and MNTD of the surveys with 1 N-fixer to 0. The model for MNTD with multiple N-fixers on both baseline survey and last resurvey excludes 1 outlier.

	All surveys			Surveys with ≥ 2 N-fixers		
**Variable**	**MLE**	**Std. Error**	**P-value**	**MLE**	**Std. Error**	**P-value**
**Faith's PD (df = 417)**				**(df = 92)**		
**Intercept**	*-25.470*	*5.747*	*<0.001*	*30.676*	*11.413*	*0.009*
**Nitrogen accumulation**	2.796	5.910	0.636	17.017	11.685	0.149
**Δ Temperature**	1.075	4.261	0.801	9.577	7.366	0.197
**Δ Aridity**	*16.500*	*7.587*	*0.030*	*47.885*	*21.702*	*0.030*
**Δ Cover**	-3.007	2.633	0.254	2.191	6.060	0.719
**Baseline Faith's PD**	*-60.748*	*2.758*	*<0.001*	-64.971	4.956	<0.001
**Marginal R^2**	0.555			0.600		
**Conditional R^2**	0.615			0.637		
**MPD (df = 417)**				**(df = 92)**		
**Intercept**	*-11.444*	*4.195*	*0.007*	*22.335*	*6.223*	*0.001*
**Nitrogen accumulation**	1.548	4.258	0.716	5.777	6.266	0.359
**Δ Temperature**	1.036	3.080	0.737	6.414	4.018	0.114
**Δ Aridity**	*10.625*	*5.380*	*0.049*	14.810	11.687	0.208
**Δ Cover**	-2.799	1.804	0.122	2.468	3.416	0.472
**Baseline MPD**	*-37.277*	*1.858*	*<0.001*	*-33.981*	*3.438*	*<0.001*
**Marginal R^2**	0.489			0.474		
**Conditional R^2**	0.573			0.509		
**MNTD (df = 417)**				**(df = 91)**		
**Intercept**	*-7.934*	*3.743*	*0.035*	*17.520*	*5.728*	*0.003*
**Nitrogen accumulation**	1.309	3.794	0.730	4.295	6.084	0.482
**Δ Temperature**	0.667	2.748	0.808	4.303	3.850	0.267
**Δ Aridity**	8.214	4.783	0.087	6.166	11.072	0.579
**Δ Cover**	-3.072	1.603	0.056	2.447	3.118	0.435
**Baseline MNTD**	*-35.251*	*1.643*	*<0.001*	*-29.721*	*3.147*	*<0.001*
**Marginal R^2**	0.506			0.438		
**Conditional R^2**	0.589			0.498		

Table S16.

Predicted effects of baseline conditions and changes of nitrogen deposition, temperature, and aridity on cubic-root transformed N-fixer PD. The full model includes the site as a random effect for intercept. We set MPD and MNTD of the surveys with 1 N-fixer to 0. The model for MNTD with >1 N-fixer in 1 or more surveys excluded 1 outlier.

	All surveys		
**Variable**	**MLE**	**Std. Error**	**P-value**
**Faith's PD (df = 417)**			
**Intercept**	*-1.220*	*0.284*	*<0.001*
**Nitrogen accumulation**	-0.116	0.291	0.690
**Δ Temperature**	0.058	0.210	0.782
**Δ Aridity**	*0.745*	*0.373*	*0.046*
**Δ Cover**	-0.203	0.128	0.114
**Baseline Faith's PD**	*-2.093*	*0.134*	*<0.001*
**Marginal R^2**	0.396		
**Conditional R^2**	0.483		
**MPD (df = 417)**			
**Intercept**	*-0.745*	*0.269*	*0.006*
**Nitrogen accumulation**	0.021	0.275	0.938
**Δ Temperature**	-0.019	0.199	0.925
**Δ Aridity**	0.679	0.349	0.052
**Δ Cover**	-0.232	0.119	0.051
**Baseline MPD**	*-1.741*	*0.122*	*<0.001*
**Marginal R^2**	0.334		
**Conditional R^2**	0.437		
**MNTD (df = 416)**			
**Intercept**	*-1.263*	*0.256*	*<0.001*
**Nitrogen accumulation**	-0.180	0.269	0.503
**Δ Temperature**	-0.001	0.192	0.997
**Δ Aridity**	0.395	0.351	0.260
**Δ Cover**	-0.250	0.133	0.061
**Baseline MNTD**	*-1.836*	*0.138*	*<0.001*
**Marginal R^2**	0.315		
**Conditional R^2**	0.373		

Table S17.

Predicted effects of nitrogen deposition, temperature, and aridity on the change of the ratio of forest N-fixer PD and overall forest PD. The full model includes the site as a random effect for intercept. We set MPD and MNTD of the surveys with 1 N-fixer to 0. The model for MNTD with multiple N-fixers on both baseline survey and last resurvey excludes 1 outlier.

	All surveys			Surveys with ≥ 2 N-fixers		
**Variable**	**MLE**	**Std. Error**	**P-value**	**MLE**	**Std. Error**	**P-value**
**Faith's PD (df = 417)**				**(df = 92)**		
**Intercept**	*-0.007*	*0.002*	*<0.001*	*0.012*	*0.004*	*0.002*
**Nitrogen accumulation**	0.002	0.002	0.228	*0.012*	*0.004*	*0.003*
**Δ Temperature**	0.000	0.001	0.841	0.001	0.002	0.636
**Δ Aridity**	*0.007*	*0.003*	*0.008*	*0.019*	*0.007*	*0.011*
**Δ Cover**	0.000	0.001	0.698	0.003	0.002	0.197
**Baseline Faith's PD**	*-0.023*	*0.001*	*<0.001*	*-0.024*	*0.002*	*<0.001*
**Marginal R^2**	0.580			0.613		
**Conditional R^2**	0.612			0.613		
**MPD (df = 417)**				**(df = 92)**		
**Intercept**	*-0.040*	*0.016*	*0.014*	*0.096*	*0.025*	*<0.001*
**Nitrogen accumulation**	0.008	0.017	0.652	0.034	0.025	0.170
**Δ Temperature**	0.004	0.012	0.721	0.028	0.016	0.079
**Δ Aridity**	*0.042*	*0.021*	*0.046*	0.064	0.046	0.169
**Δ Cover**	-0.010	0.007	0.149	0.008	0.014	0.569
**Baseline MPD**	*-0.144*	*0.007*	*<0.001*	*-0.133*	*0.014*	*<0.001*
**Marginal R^2**	0.494			0.462		
**Conditional R^2**	0.573			0.485		
**MNTD (df = 417)**				**(df = 91)**		
**Intercept**	-0.070	0.039	0.068	*0.150*	*0.058*	*0.011*
**Nitrogen accumulation**	0.018	0.039	0.638	0.082	0.060	0.178
**Δ Temperature**	-0.010	0.028	0.729	0.010	0.038	0.798
**Δ Aridity**	0.086	0.049	0.078	0.056	0.109	0.608
**Δ Cover**	-0.030	0.016	0.061	0.027	0.030	0.375
**Baseline MNTD**	*-0.299*	*0.016*	*<0.001*	*-0.243*	*0.027*	*<0.001*
**Marginal R^2**	0.461			0.414		
**Conditional R^2**	0.560			0.489		

Table S18.

Predicted effects of nitrogen deposition, temperature, and aridity on the cubic-root transformed change of the ratio of forest N-fixer PD and overall forest PD. The full model includes the site as a random effect for intercept. We set MPD and MNTD of the surveys with 1 N-fixer to 0. The models of MNTD, as well as those of PD with multiple N-fixers on both baseline survey and last resurvey did not need to be transformed. The model for Faith's PD excluded 1 outlier.

	All surveys		
**Variable**	**MLE**	**Std. Error**	**P-value**
**Faith's PD (df = 416)**			
**Intercept**	*-0.070*	*0.020*	*<0.001*
**Nitrogen accumulation**	-0.002	0.020	0.914
**Δ Temperature**	0.006	0.015	0.695
**Δ Aridity**	*0.055*	*0.026*	*0.037*
**Δ Cover**	-0.008	0.009	0.404
**Baseline Faith's PD**	*-0.148*	*0.009*	*<0.001*
**Marginal R^2**	0.409		
**Conditional R^2**	0.483		
**MPD (df = 417)**			
**Intercept**	*-0.104*	*0.043*	*0.016*
**Nitrogen accumulation**	0.007	0.044	0.881
**Δ Temperature**	0.004	0.032	0.891
**Δ Aridity**	*0.114*	*0.056*	*0.041*
**Δ Cover**	-0.036	0.019	0.059
**Baseline MPD**	*-0.265*	*0.019*	*<0.001*
**Marginal R^2**	0.321		
**Conditional R^2**	0.422		
**MNTD (df = 417)**			
**Intercept**	*-0.138*	*0.056*	*0.015*
**Nitrogen accumulation**	0.013	0.058	0.818
**Δ Temperature**	-0.028	0.042	0.505
**Δ Aridity**	0.137	0.073	0.063
**Δ Cover**	-0.043	0.025	0.086
**Baseline MNTD**	*-0.328*	*0.025*	*<0.001*
**Marginal R^2**	0.309		
**Conditional R^2**	0.413		

Table S19.

Estimated effects of nitrogen deposition, temperature, and aridity on the abundance of forest N-fixers. The full model includes the site as a random effect for intercept.

Variable	MLE	Std. Error	P-value
**Intercept**	*12.313*	*2.448*	*<0.001*
**Nitrogen deposition**	*-0.135*	*0.036*	*<0.001*
**Δ Temperature**	-1.432	1.350	0.289
**Δ Aridity**	-12.114	10.409	0.245
**Baseline N-fixer cover**	*-0.886*	*0.026*	*<0.001*
**Marginal R^2**	0.697		
**Conditional R^2**	0.779		

Table S20.

Estimated effects of nitrogen deposition, temperature, and aridity on the cubic-root transformed abundance of forest N-fixers. The full model includes the site as a random effect for intercept, and excludes 5 outliers.

Variable	MLE	Std. Error	P-value
**Intercept**	*1.330*	*0.338*	*<0.001*
**Nitrogen deposition**	*-0.015*	*0.005*	*0.002*
**Δ Temperature**	-0.346	0.183	0.059
**Δ Aridity**	0.409	1.423	0.774
**Baseline N-fixer cover**	*-0.074*	*0.003*	*<0.001*
**Marginal R^2**	0.469		
**Conditional R^2**	0.636		

Table S21.

Estimated effects of nitrogen deposition, temperature, and aridity on the proportion of forest abundance from N-fixer species. The full model includes the site as a random effect for intercept.

Variable	MLE	Std. Error	P-value
**Intercept**	*0.157*	*0.028*	*<0.001*
**Nitrogen deposition**	*-0.002*	*0.000*	*<0.001*
**Δ Temperature**	-0.020	0.015	0.184
**Δ Aridity**	-0.174	0.118	0.139
**Baseline N-fixer cover proportion**	*-0.865*	*0.021*	*<0.001*
**Marginal R^2**	0.728		
**Conditional R^2**	0.803		

Table S22.

Estimated effects of nitrogen deposition, temperature, and aridity on the cubic-root transformed proportion of forest abundance from N-fixer species. The full model includes the site as a random effect for intercept, and excludes 1 outlier.

Variable	MLE	Std. Error	P-value
**Intercept**	*0.277*	*0.078*	*<0.001*
**Nitrogen deposition**	*-0.003*	*0.001*	*0.006*
**Δ Temperature**	-0.065	0.044	0.137
**Δ Aridity**	0.066	0.334	0.843
**Baseline N-fixer cover proportion**	*-1.446*	*0.073*	*<0.001*
**Marginal R^2**	0.404		
**Conditional R^2**	0.544		
